# Nuggets

**Published:** 1891-01

**Authors:** C. Carleton Smith

**Affiliations:** Philadelphia


### NUGGETS.

C. CARLETON SMITH, M. D., PHILADELPHIA.

Intense dryness of the mouth and fauces, so dry as to cause choking, especially on awaking in the morning, is often a symptom of the coming on of a serious attack of illness. And patients thus suffering have no thirst whatever, but they run for water as soon as they awake to moisten the parts. We have six drugs which produce this morbid condition, viz.: Paris-quad., Dioscorea vill., Lycopod., Nux-mos., Puls., and Sulphur.

Paris-quad. has great dryness of the tongue and mouth when waking from sleep at any time; tongue coated white, with roughness of the surface, bitter or altered taste, and no thirst. Dioscorea has very dry tongue in the morning with heavy brown coating, bitter taste, and no thirst.

Lycopod. has dry tongue in the morning with great stiffness of the organ, generally free from coating, bitter, fatty, saltish, or sour taste.

Nux-mos. has continual dryness, with a paralyzed condition of the tongue, and entire absence of thirst or taste.

Pulsatilla has dry tongue, as if burnt, which makes it feel insensible in the morning. Grayish coating of tough, tenacious mucus, without thirst, and with earthy, flat, but more especially bitter, putrid saltish, sour taste.

Sulphur has dry, brown, parched, rough tongue in the morning *with thirst*, and with either bitter, flat, putrid, saltish, or more frequently *sour* taste. When you hear a patient who is becoming seriously ill complain of "feeling of *great weight in the nape of the neck*," think first of Paris-quad.

When the baby cries all day long and sleeps soundly all night study Lycopod. And tell the nurse to save the baby's napkin after it has been urinated upon—lay it away on a smooth surface to dry—look at it with a magnifying glass, and you will generally find fine particles of beautiful red sand. Lyc. will set the matter all right.

Persons recovering from a severe attack of pneumonia will frequently call your attention to the fact that their lungs are not right, they are *full* of *smoke*, and that they smell pine smoke as if wood was burning. Give such patients a dose or two of Baryta-carb., high. If they complain of *paper* burning, think of Coffea. A *constant* tormenting, urging in the rectum without a stool; wanting to pass a stool, but the constant pain increased by urging, and the patient is obliged to desist, Lachesis.

A most harassing titillating cough in children at night as soon as their heads touch the pillow, but not at all in day-time. Drosera will probably help you out.

Conium is also one of our best remedies for cough with aggravation as soon as head touches pillow. But it has a cough which is very troublesome through the day.

It is a well-known fact that patients suffering with dyspnœa from whatever condition of the lungs or bronchia are, as a rule, made worse by lying down. But when a case is met with which has "gasping for breath as soon as he assumes the sitting posture," think at once of Laurocerasus.

In this latitude we frequently find Rhus-tox. and Rhododendron indicated in rheumatic conditions. And in their use it is well to bear in mind these distinguishing marks: Under Rhod. pains do not admit of the limbs being at rest, a desire to move and moving relieves; Rhus occasions uneasiness in the painful parts, but on moving the pains are worse, continued motion only relieves.

Rhod. has general aggravation of pains before a change in the weather, particularly before a thunder-storm. And Dr. Hering said even in dysentery this holds true. Rhus has aggravation from the warmth of the bed or from getting wet while perspiring.

Rhod. acts more particularly on the right side of body, and Rhus on the left.

If you observe as a marked characteristic symptom in a case,

"*quivering* of *the* LEFT *upper eyelid*," especially in convulsions of children, give Arum-triphyllum.. Where a sharp fish-bone has wounded the œsophagus, give Cicuta-virosa.

When the sensation of a fish-bone remains in throat, and yet you cannot discover any, give Hepar.

When the œsophagus has been burned by swallowing hot potato or hot drinks, give Sapo soda$^{30}$. In case of stools pouring away from the anus, feeling as hot as boiling water, Mercurius-sulphuricus is the remedy. The next similar drug to this is Calc-phos., which has daily, watery, very hot stools. Acute pain in the eye after a blow from a blunt instrument, or from a baby's fist, with spasmodic closing of the lid, with feeling as if lid slipped over a round smooth lump, calls for Symphytum-off. Bearing-down pains, with pressure on the bladder and frequent desire to urinate, all relieved by horseback riding, Lycopod. is the remedy.

We meet with young people, sometimes, whose hair has fallen out in spots and those spots have become gray when the hair is renewed. Such cases need Vinca-minor.

Patients who cannot sit at all because their backs ache so intensely, accompanied with burning along the whole spine, give Zincum. Also compare Cobalt., Puls., and Sepia.

In a case of nephralgia or urinary calculi occurring on the right side, and you are in doubt whether to give Lyc. or Berberis, if the patient should have been before the attack, or is, at the time, suffering with acute rheumatism of knee-joint on that side, Berberis will be the preferable remedy.

Colocynthis and Staphisagria are very similar, not only in their anger and inclination to be sorely vexed, but also in regard to the abdominal colic, neuralgia, and dysentery, and hence should be studied together. Arsenicum, drinks little, but often. Bryonia, drinks much but not often. Bry., eats often, but little at a time. Ars., much eating at one time.

Do not overlook the fact that the Lachesis aggravation does not occur after sleep. On the contrary, the Lach. patient sleeps *into* the aggravation which awakes him; this occurs with each nap. But aggravation after a full and complete sleep we have under Kali-bich. And both these drugs have sensitiveness of the throat to touch or contact of clothing, but only the former in an exquisite degree.

